# Soluble Dipeptidyl Peptidase-4 Induces Fibroblast Activation Through Proteinase-Activated Receptor-2

**DOI:** 10.3389/fphar.2020.552818

**Published:** 2020-09-30

**Authors:** Shih-Yi Lee, Shao-Tung Wu, Yao-Jen Liang, Ming-Jai Su, Cheng-Wei Huang, Yu-Hsuan Jao, Hui-Chun Ku

**Affiliations:** ^1^ Division of Pulmonary and Critical Care Medicine, MacKay Memorial Hospital, Taipei, Taiwan; ^2^ MacKay Junior College of Medicine, Nursing and Management, Taipei, Taiwan; ^3^ Division of Pulmonary and Critical Care Medicine, Taitung MacKay Memorial Hospital, Taitung, Taiwan; ^4^ Department of Life Science, Fu Jen Catholic University, New Taipei City, Taiwan; ^5^ College of Medicine, Institute of Pharmacology, National Taiwan University, Taipei, Taiwan

**Keywords:** dipeptidyl peptidase-4, fibrosis, fibroblast, proteinase-activated receptor-2, nuclear factor-kappa B, suppressor of mothers against decapentaplegic

## Abstract

Fibroblasts are the chief secretory cells of the extracellular matrix (ECM) responsible for basal deposition and degradation of the ECM under normal conditions. During stress, fibroblasts undergo continuous activation, which is defined as the differentiation of fibroblasts into myofibroblasts, a cell type with an elevated capacity for secreting ECM proteins. Dipeptidyl peptidase-4 (DPP4) is a ubiquitously expressed transmembrane glycoprotein and exerts effects that are both dependent and independent of its enzymatic activity. DPP4 has been demonstrated to define fibroblast populations in human skin biopsies of systemic sclerosis. Shedding of DPP4 from different tissues into the circulation appears to be involved in the pathogenesis of the diseases. The mechanism underlying soluble DPP4–induced dermal fibrosis has not been clearly determined. The effects of DPP4 on murine 3T3 fibroblasts and human dermal fibroblasts were evaluated by measuring the expression of fibrotic proteins, such as α-SMA and collagen. Soluble DPP4 stimulated the activation of fibroblasts in a dose-dependent manner by activating nuclear factor-kappa B (NF-κB) and suppressor of mothers against decapentaplegic (SMAD) signaling. Blocking proteinase-activated receptor-2 (PAR2) abrogated the DPP4-induced activation of NF-κB and SMAD and expression of fibrosis-associated proteins in fibroblasts. Linagliptin, a clinically available DPP4 inhibitor, was observed to abrogate the soluble DPP4–induced expression of fibrotic proteins. This study demonstrated the mechanism underlying soluble DPP4, which activated NF-κB and SMAD signaling through PAR2, leading to fibroblast activation. Our data extend the current view of soluble DPP4. Elevated levels of circulating soluble DPP4 may contribute to one of the mediators that induce dermal fibrosis in patients.

## Introduction

Fibrotic disorders encompass a wide spectrum of clinical entities such as systemic sclerosis, a systemic fibrotic disease that induces fibrosis of the skin and internal organs (Rosenbloom et al., 2017). Fibrotic disorders involve a complex and multistage process of tissue injury and inflammation (Lee and Kalluri, 2010). This process is constituted by extracellular matrix (ECM) expansion that is orchestrated by a network of cytokines, chemokines, growth factors, adhesion molecules, and signaling transduction processes (Lee and Kalluri, 2010). Fibroblasts are the chief secretory cells of the ECM (Biernacka and Frangogiannis, 2011). They remain quiescent under normal conditions and are responsible for the basal deposition and degradation of the ECM as well as the maintenance of the matrix network (Krenning et al., 2010). Under stress, stimulated by mediators released from injured and inflammatory tissue, fibroblasts undergo continuous activation, which is defined as the differentiation of fibroblasts into myofibroblasts (Kendall and Feghali-Bostwick, 2014), a cell type with an elevated capacity for secreting ECM proteins. TGF-β is one of the crucial mediators (Walton et al., 2017), which activates suppressor of mothers against decapentaplegic (SMAD), mitogen-activated protein kinase (MAPK), and nuclear factor-kappa B phosphorylated (NF-κB) signaling to induce the pathogenesis of fibrosis (He and Dai, 2015). Myofibroblasts are ultimately responsible for the replacement of healthy tissues with nonfunctional fibrotic tissues (McAnulty, 2007), which leads to increased tissue stiffness and ultimately organ failure (Tomasek et al., 2002). Because of the lack of effective therapeutic agents and insufficient knowledge regarding their pathogenesis in fibrotic diseases, identification of the mediator that regulates fibroblast activation and differentiation of fibroblasts into myofibroblasts is urgently required.

Dipeptidyl peptidase-4 (DPP4), also known as CD26 (Morimoto and Schlossman, 1998), is a type II transmembrane glycoprotein expressed in various cell types that has multifunctional properties (Rohrborn et al., 2015). DPP4 inhibitors, also commonly called gliptin, are being developed as a class of drugs for treating diabetes (Neumiller et al., 2010). In addition to its enzymatic activity, DPP4 itself participates in other cellular functions. Change in DPP4 expression is associated with disease progression (Trzaskalski et al., 2020). Increased DPP4 expression and activity have demonstrated an association with inflammation observed in obesity and metabolic disorders (Trzaskalski et al., 2020). DPP4 is highly expressed in bronchial epithelial cells of patients with asthma, and it increased cell proliferation in airway constitutive cells (Shiobara et al., 2016). Increased DPP4 levels were observed in fibroblasts isolated from individuals with systemic sclerosis relative to fibroblasts isolated from healthy individuals (Soare et al., 2020). Furthermore, the DPP4-positive fibroblast populations in the skin are highly proliferative and expand upon tissue injury to promote wound healing (Rinkevich et al., 2015). The action of DPP4 is complicated.

DPP4 may be released through a nonclassical secretory mechanism from the membrane that involves proteolytic cleavage near the flexible region for generating the soluble form (Rohrborn et al., 2014; Nargis and Chakrabarti, 2018). Soluble DPP4 has also been suggested to be a novel regulator, and elevated levels are indicative of several disorders in addition to diabetes, such as obesity, cardiovascular disease, and nonalcoholic fatty liver disease (dos Santos et al., 2013; Baumeier et al., 2017; Nargis and Chakrabarti, 2018). Soluble DPP4 is an adipokine (Lamers et al., 2011) and is positively associated with hemoglobin A1c levels and the insulin resistance index in type 2 diabetes (Sell et al., 2013; Nargis and Chakrabarti, 2018). It directly impairs insulin signaling in adipocytes, smooth muscle cells, and hepatocytes (Wronkowitz et al., 2014; Baumeier et al., 2017), whereas insulin-stimulated Akt phosphorylation was observed to be reduced when soluble DPP4 was administered (Baumeier et al., 2017). Soluble DPP4 also activates the MAPK and NF-κB signaling cascade involving proteinase-activated receptor-2 (PAR2), resulting in the induction of inflammation and proliferation of human vascular smooth muscle cells (Wronkowitz et al., 2014). Reports have suggested that soluble DPP4 not only possesses catalytic functions but also activates some receptors and signal pathways. Numerous reports have been published on membrane-bound DPP4; however, little information is available on soluble DPP4. The signaling pathway underlying DPP4 in the pathogenesis of fibrosis is still unclear, and revealing this mechanism could potentially lead to a greater understanding of the pathophysiology and treatments of fibrosis diseases. We hypothesized that soluble DPP4 plays a role in dermal fibrosis. Using cultured fibroblasts, we examined the mechanism of how soluble DPP4 enhances fibroblast activation.

## Material and Methods

### Cell Culture

Murine NIH/3T3 fibroblasts were purchased from the American Type Culture Collection. Cells were cultured in Dulbecco’s modified Eagle’s medium supplemented with 10% fetal bovine serum (Gibco, Scotland, UK) and antibiotics (100 μg/ml penicillin and 100 μg/ml streptomycin; Amresco, Solon, OH, USA), and incubated at 37°C under a 5% CO_2_/95% air atmosphere. Fibroblasts were seeded at 5,000 cells/cm^2^ and incubated overnight to allow attachment in a monolayer for determining the effect of soluble DPP4 on fibroblast activation. Cells were treated with either transforming growth factor-β (TGF-β; Cayman, MI, USA; 10 ng/ml) or recombinant DPP4 (Cayman, MI, USA; 80 and 200 ng/ml) for 48 h. To explore the signaling pathway of soluble DPP4, cells were incubated with an enzyme inhibitor of DPP4 (linagliptin; 30 nM), an antagonist of PAR2 (GB83; 10 μM), or an inhibitor of NF-kB (Bay11-7082; 1 μM), individually, for 30 min before administering the recombinant DPP4.

Adult normal human dermal fibroblasts (HDF) were purchased from the Bioresource Collection and Research Center (BCRC, Hsinchu, TW). HDF cells were maintained in Dulbecco’s modified Eagle’s medium supplemented with 15% fetal bovine serum (Gibco, Scotland, UK) and antibiotics (100 μg/ml penicillin and 100 μg/ml streptomycin; Amresco, Solon, OH, USA), and incubated at 37°C under a 5% CO2/95% air atmosphere. HDF were starved in DMEM for 24 h and then treated with TGF-β (Cayman, MI, USA; 10 ng/ml) or recombinant DPP4 (Cayman, MI, USA; 200 ng/ml) for another 48 h.

### Detection of Cell Viability

MTT [3-(4,5-Dimethylthiazol-2-yl)-2,5-diphenyltetrazoliumbromide] assay was used as the assessment of cell viability. MTT was then added in cell culture in a final concentration of 0.5 mg/ml. After discarding the supernatant, the purple formazan crystals were dissolved in DMSO. Solutions were then loaded in a 96-well plate, and determined on an automated microplate spectrophotometer at 570 nm.

### Protein Extraction From Cell Culture

The proteins from cell culture were extracted using RIPA buffer (Thermo Fisher Scientific Inc., IL, USA) containing protease and phosphatase inhibitors (Sigma, St. Louis, MO, USA). A BCA protein assay kit (Thermo Fisher Scientific Inc.) was used to determine the protein concentration.

### Determination of Protein Expression

A Western blotting technique was performed to detect protein expression. Equal quantities of proteins were first denatured for 10 min in boiling sample buffer (31.3 mM Tris-HCl at pH 6.8, 25% glycerol, 10% sodium dodecyl sulfate (SDS), 10% 2-mercaptoethanol, and 0.00125% bromophenol blue). Then, proteins were separated using SDS–polyacrylamide gel electrophoresis and transferred to polvinylidene difluoride membranes (Perkin-Elmer Life Sciences, Boston, MA, USA). The membranes were blocked with 5% fat-free milk dissolved in Tris-buffered saline with Tween 20 (TBST) and incubated overnight with the primary antibodies of collagen I (1:1000 dilution; Abcam, USA), elastin (1:1000 dilution; Abcam, USA), α-smooth muscle actin (α-SMA; 1:1000 dilution; Abcam, USA), phosphorylated and total extracellular signal–regulated kinase (p-ERK and ERK; 1:1000 dilution; Cell Signaling, USA), NF-κB p65 and p-p65 (1:1000 dilution; Cell Signaling, USA), SMAD2/3 and p-SMAD2/3 (1:1000 dilution; Abcam, USA), and glyceraldehyde 3-phosphate dehydrogenase (GAPDH; 1:1000 dilution; Santa Cruz Biotechnology, CA, USA) at 4°C. Subsequently, the membranes were washed three times for 15 min each with TBST, which was followed by incubation with horseradish-peroxidase-conjugated secondary antibodies (Santa Cruz Biotechnology, Inc.) for 1 h. After washing three times for 20 min each with TBST, the protein signals were detected using an enhanced chemiluminescence system (Millipore, Bedford, MA, USA). The blots were scanned and quantified using Imagequant (Molecular Dynamics, Inc., Sunnyvale, CA, USA).

### RNA Extraction and Reverse Transcription Quantitative Polymerase Chain Reaction

Total RNA was isolated from cells using TRIzol (Thermo Fisher Scientific, MA, USA). Total RNA was reverse transcribed with Maxima First Strand cDNA Synthesis Kit (Thermo Fisher Scientific, MA, USA) and SYBR Green was used for performing quantitative real time PCR. The following are the sequences of the primers used for amplification. *PAR2*: CGCACTGTAAAGCAGATGCAA and AATTCCCATCTGAGGACCTGG; *ACTA2*: GCCTGAGGGAAGGTCCTAA and GGAGCTGCTTCACAGGATTC; *COL1A1*: CACAGAGGTTTCAGTGGTTTGG and AGTAGCACCATCATTTCCACGA; *HPRT*: CGTCTTGCTCGAGATGTGATG and GCACACAGAGGGCTACAATGTG.

DNA was amplified for 40 cycles of denaturation for 5 s at 95°C and annealing for 30 s at 60°C, using the TaKaRa Thermal Cycler Dice (TP900). The qPCR assays were performed and analyzed using the Thermal Cycler Dice Real Time System version 4.2 (TaKaRa). The expression level of each individual transcript was normalized to HPRT gene and expressed relative to the mean expression values of control samples.

### Immunofluorescent Staining

The expressions of α-SMA, p-p65, and p-SMAD in cells were analyzed by immunofluorescence staining. Cells were cultured on glass coverslips at the density of 3,000 cells/cm^2^ and treated with DPP4 with or without GB83 or linagliptin for 48 h. Following treatment for 48 h, cells in the basement layer were washed with PBS and fixed in 4% paraformaldehyde. Following three times washes with PBS for 5 min each, the cells were treated with 0.5% Triton X-100 for 10 min and blocked with bovine serum albumin (Sigma-Aldrich; Merck KGaA) for 1 h at room temperature, and subsequently incubated with α-SMA antibodies (1:100 dilution; Abcam, USA) at 4°C overnight. Cells were washed three times for 5 min each with PBS and then incubated with secondary antibodies (1:200 dilution; Abcam, USA) for another 1 h. Following washing three times for 5 min each with PBS, cells were stained with DAPI (Beyotime Institute of Biotechnology) at room temperature to visualize the nuclei. Following washing with PBS, the slide was mounted with anti-fluorescence quenching agent (Abcam, USA) and cover-slipped; digital images were captured using an inverted fluorescent microscope (magnification, ×400). Digital images were captured four fields per sample with the same exposure time. Three independent experiments were performed.

### Lentiviral Transduction for Gene Knockdown

Lentiviral particles containing shRNAs pLKO.1 (#TRCN0000006770) was used to knockdown PAR2 in human dermal fibroblasts. Lentivirus containing scrambled shRNA, pLKO-shScr (#TRCN00001) was used as non-targeting control and served as wild-type. Human adult fibroblasts were transduced with lentiviral vectors with MOI of 3, along with 5 ug/ml polybrene (Sigma Aldrich, MO, USA. Transduced human dermal fibroblasts were treated with puromycin (2 ug/ml), for selection of transduced cells.

### Statistical Analyses

All values are represented as mean ± standard error. The results were analyzed using one-way ANOVA, followed by Bonferroni *post hoc* tests. We considered that p < 0.05 to be significant.

## Results

### Soluble DPP4 Enhanced Fibroblast Activation

**Figure 1:**
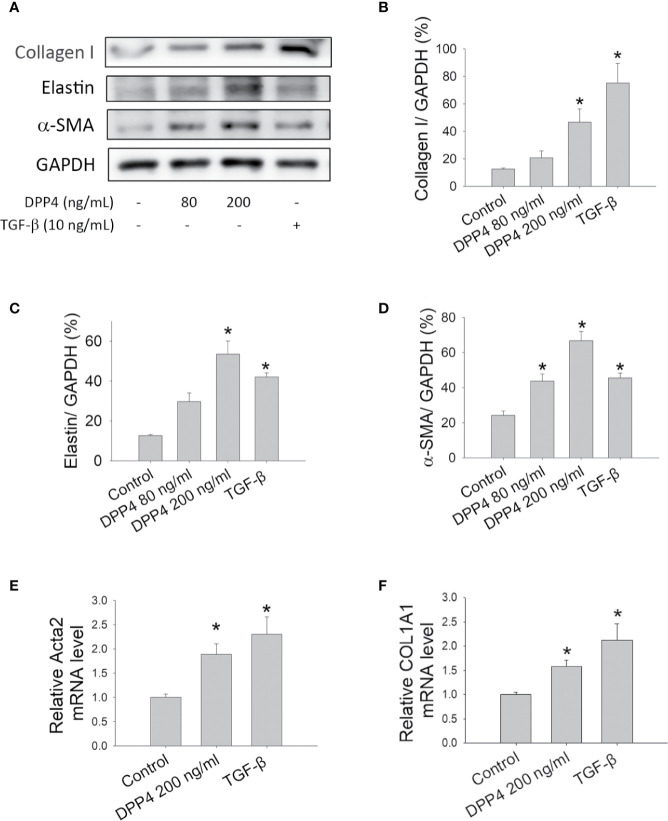
Effects of soluble DPP4 on the activation of fibroblasts. Protein expression measured in fibroblasts after their treatment with recombinant DPP4 (80 and 200 ng/ml) or TGF-β (10 ng/ml) for 48 h. **(A)** Protein expression in NIH-3T3 fibroblasts was determined using Western blotting. Calculated ratios of **(B)** collagen I to GAPDH, **(C)** elastin to GAPDH, and **(D)** α-SMA to GAPDH were shown. **(E)** *ACTA2*, and **(F)** *COL1A1* mRNA expressions were measured in human dermal fibroblast by using RT-qPCR. (n = 4) *p < 0.05 vs. control.

Fibroblasts are key effector cells in tissue fibrosis (Krenning et al., 2010). TGF-β has been well recognized as a fibrotic cytokine that activates fibroblasts and drives their differentiation into myofibroblasts, leading to organ fibrosis. To determine the effect of soluble DPP4 on the pathogenesis of fibrosis, murine 3T3 fibroblasts were treated with recombinant DPP4 (80 and 200 ng/ml) or TGF-β (10  ng/ml) for 48  h and examined for the expression of fibrotic markers. Administration of TGF-β served as a positive control. Compared with control groups, DPP4 increased the expression of fibrotic markers (**Figure 1A**), such as collagen I (**Figure 1B**), elastin (**Figure 1C**), and α-SMA (**Figure 1D**), in a concentration-dependent manner, indicating that soluble DPP4 promoted the activation and transformation of fibroblasts into differentiated myofibroblasts.

Skin biopsies from patients with systemic sclerosis were highlighted by a prominent increase in the expression of ECM transcripts (Chadli et al., 2019). Activated dermal fibroblasts are considered to play a major role in the development of skin fibrosis in systemic sclerosis (Chadli et al., 2019). To confirm the results in human dermal fibrotic disease, primary human dermal fibroblasts were treated with DPP4 (200 ng/ml) or TGF-β (10 ng/ml) for 48 h. Both DPP4 and TGF-β increased the expression of fibrotic gene expression, including *ACTA2* (**Figure 1E**) and *COL1A1* (**Figure 1F**), confirming the role of soluble DPP4 in human fibrotic disease.

### Soluble DPP4 Activated the Transcription Factor Signaling Pathway in Fibroblasts

**Figure 2:**
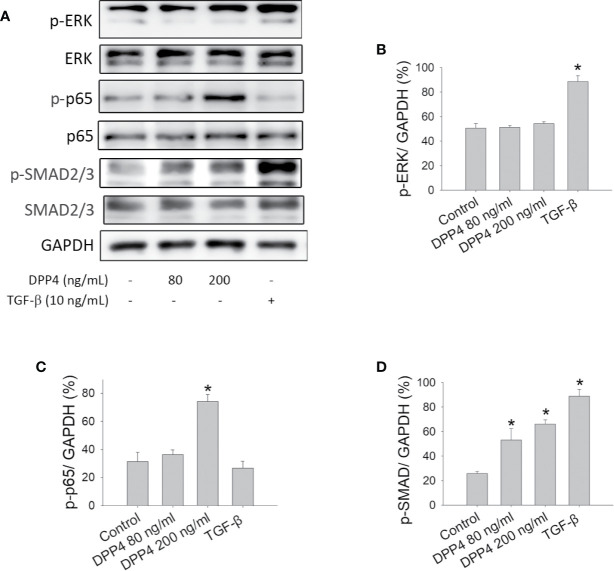
Effects of soluble DPP4 on protein kinases and transcription factors in fibroblasts. Protein expression measured in NIH-3T3 fibroblasts after their treatment with recombinant DPP4 (80 and 200 ng/ml) or TGF-β (10 ng/ml) for 48 h. **(A)** Protein expression determined using Western blotting. Calculated ratios of **(B)** p-ERK to GAPDH, **(C)** p-p65 to GAPDH, and **(D)** p-SMAD2/3 to GAPDH were shown. (n = 4) *p < 0.05 vs. control.

To determine the signaling pathway involved in soluble DPP4–induced fibrosis and to compare it with TGF-β-induced signaling, we investigated the effect of soluble DPP4 and TGF-β on protein kinase and transcription factor in murine 3T3 fibroblasts. In our study, after 48 h of DPP4 or TGF-β exposure, only TGF-β but not DPP4 induced ERK phosphorylation (**Figures 2A, B**). By contrast, DPP4 enhanced the expression of NF‐κB p-p65 (**Figures 2A, C**). In addition, both soluble DPP4 and TGF-β increased the expression of p-SMAD2/3 (**Figures 2A, D**). Soluble DPP4 induced a complex signaling pathway in the induction of the fibrotic signaling pathway.

### NF-κB Stimulated by Soluble DPP4 Led to Fibroblast Activation

**Figure 3:**
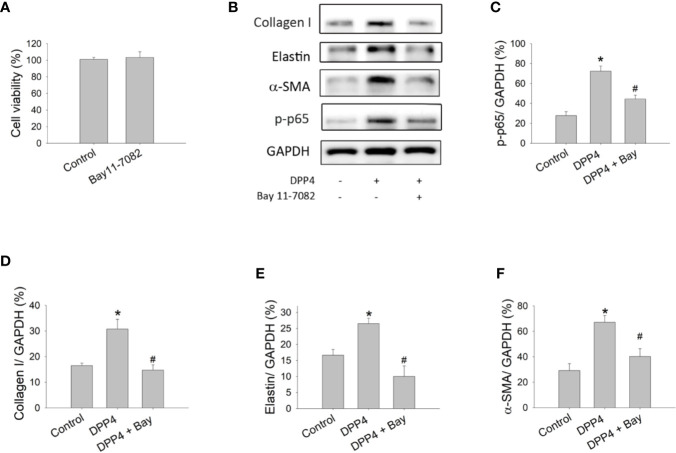
Effects of NF-κB inhibition on the activation of fibroblasts. Cell viability and protein expression were measured in NIH-3T3 fibroblasts after their treatment with recombinant DPP4 (200 ng/ml) in the presence or absence of Bay11-7082 (1 µM), a NF-κB inhibitor. **(A)** Cell viability of fibroblasts exposed to Bay11-7082. **(B)** Protein expression determined using Western blotting. Calculated ratios of **(C)** p-p65 to GAPDH, **(D)** collagen I to GAPDH, **(E)** elastin to GAPDH, and **(F)** α-SMA to GAPDH were shown. (n = 4) *p < 0.05 vs. control. ^#^ *p* < 0.05 vs. DPP4.

To confirm the role of NF-κB in soluble DPP4 signaling, an NF-κB inhibitor, Bay11-7082, was used in murine 3T3 fibroblasts. Bay11-7082 at a concentration of 1 μM had no effect on the cell viability of fibroblasts (**Figure 3A**); however, it inhibited the DPP4-induced NF-κB p-p65 expression (**Figures 3B, C**). Furthermore, NF-κB inhibition could completely abolish the soluble DPP4–induced expression of fibrosis-associated proteins (**Figure 3B**), such as collagen I (**Figure 3D**), elastin (**Figure 3E**), and α-SMA (**Figure 3F**).

### The PAR2 Antagonist GB83 Abrogated the Soluble DPP4–Induced Response in Fibroblasts

**Figure 4:**
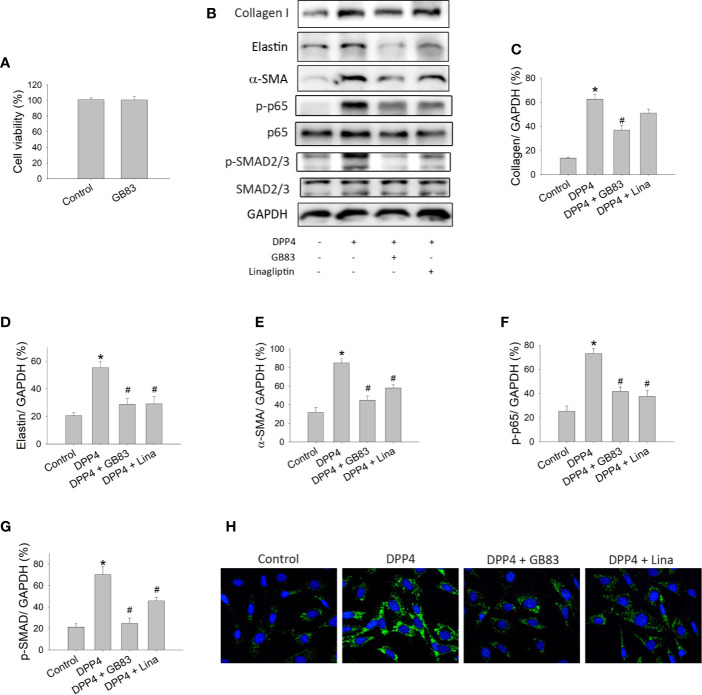
Effects of PAR2 antagonist or DPP4 inhibition on the activation of fibroblasts. Cell viability and protein expression measured in NIH-3T3 fibroblasts after their treatment with recombinant DPP4 (200 ng/ml) in the presence or absence of GB83 (10 µM) or linagliptin (30 nM). **(A)** Cell viability of fibroblasts exposed to GB83. **(B)** Protein expression determined using Western blotting. Calculated ratios of **(C)** collagen I to GAPDH, **(D)** elastin to GAPDH, **(E)** α-SMA to GAPDH, **(F)** NF-κB p-p65 to GAPDH, and **(G)** p-SMAD2/3 to GAPDH were shown. (n = 4) **(H)** Immunofluorescent staining of α-SMA. *p < 0.05 vs. control. ^#^ *p* < 0.05 vs. DPP4.

PAR2 is a seven-transmembrane domain G protein–coupled receptor that is widely expressed in cells and regulates a variety of physiological and pathophysiological processes including fibrosis (Soh et al., 2010). To determine whether the DPP4-induced fibroblast activation is associated with PAR2, murine 3T3 fibroblasts were treated with recombinant DPP4 in the presence or absence of GB83, a PAR2 antagonist. GB83 at a concentration of 10 μM did not affect the cell viability of fibroblasts and was used in the subsequent experiment (**Figure 4A**). GB83 inhibited the soluble DPP4–induced expression of fibrotic proteins (**Figure 4B**), such as collagen I (**Figure 4C**), elastin (**Figure 4D**), and α-SMA (**Figure 4E**). Furthermore, the soluble DPP4–induced activation of transcription factor signaling pathways, such as NF-κB p-p65 (**Figure 4F**) and p-SMAD2/3 pathways (**Figure 4G**), could be prevented by GB83.

### Linagliptin Prevented the Effect of Soluble DPP4 on Fibroblasts

We also investigated whether linagliptin, a clinically available enzyme inhibitor of DPP4, prevents DPP4-induced fibroblast activation. Murine 3T3 fibroblasts were treated with recombinant DPP4 in the presence or absence of linagliptin (30 nM). The concentration of linagliptin used in this study was observed to exert a protective effect that was achieved in the plasma of patients with type 2 diabetes treated with this drug (Heise et al., 2009). We observed that linagliptin abrogated the soluble DPP4–induced expression of fibrotic protein, such as elastin (**Figure 4D**) and α-SMA (**Figures 4E, H**). DPP4-induced activation of transcription factor signaling pathways, including NF-κB p-p65 (**Figure 4F**) and p-SMAD2/3 (**Figure 4G**) pathways, was also prevented by linagliptin.

### Soluble DPP4–Induced p-p65 and p-SMAD in HDF

**Figure 5:**
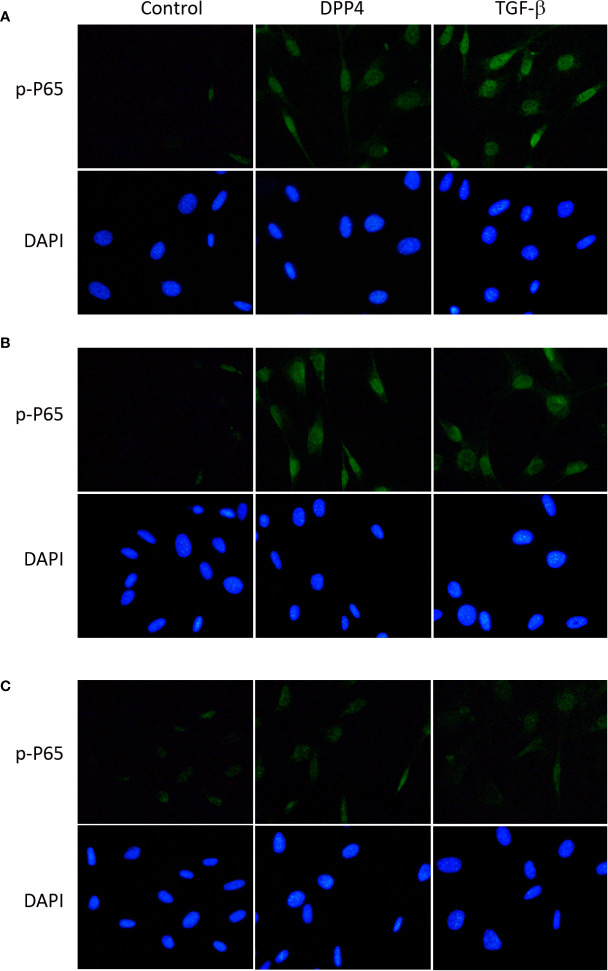
Effects of DPP4 on p-p65 activation in human dermal fibroblast. Human dermal fibroblast were treated with recombinant DPP4 (200 ng/mL) or TGF-β (10ng/mL). Immunofluorescent staining of p-p65 was determined after **(A)** 6 h, **(B)** 24 h, and **(C)** 48 h of DPP4 or TGF-β exposure.

**Figure 6:**
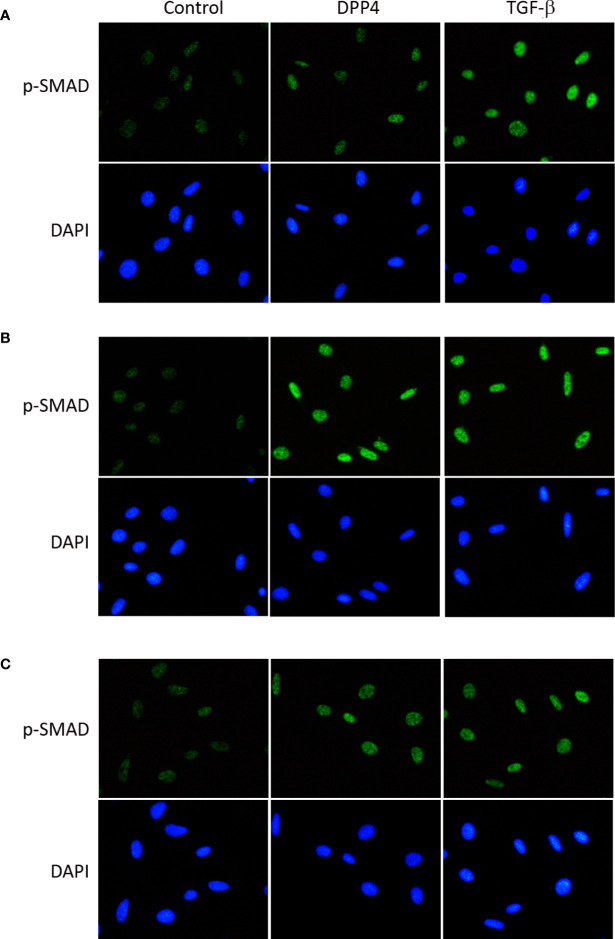
Effects of DPP4 on p-SMAD activation in human dermal fibroblast. Human dermal fibroblast were treated with recombinant DPP4 (200 ng/mL) or TGF-β (10g/mL). Immunofluorescent staining of p-SMAD was determined after **(A)** 6 h, **(B)** 24 h, and **(C)** 48 h of DPP4 or TGF-β exposure.

To determine the signaling underlying soluble DPP4 in HDF, p-p65 (**Figure 5**) and p-SMAD (**Figure 6**) were determined through immunofluorescence at different time points. Both DPP4 and TGF-β induced the activation of p-p65 and p-SMAD in 6 h and had a plateau effect in 24 h. TGF-β induced a more rapid activation than DPP4 did.

### Knocking Down PAR2 Abrogated the Soluble DPP4–Induced Response in HDF

**Figure 7:**
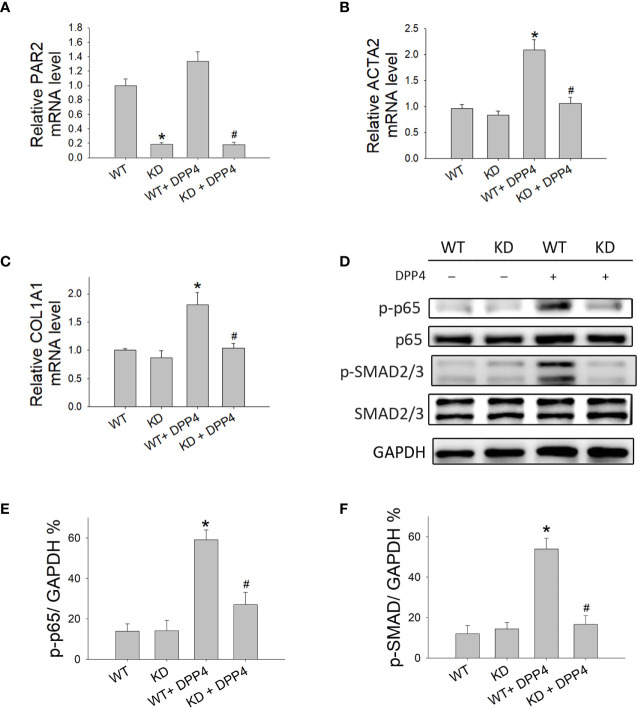
Effects of PAR2 knocked down on the activation of fibroblasts in human dermal fibroblast. Wild-type (WT) and PAR2 knockdown (KD) human dermal fibroblast were treated with recombinant DPP4 (200 ng/ml). Gene expression was determined by RT-qPCR. **(A)** PAR2, **(B)** ACTA2, and **(C)** COL1A1 mRNA expression were determined. **(D)** Protein expression was determined using Western blotting. Calculated ratios of **(E)** NF-κB p-p65 to GAPDH, **(F)** p-SMAD2/3 to GAPDH were shown. (n = 4) *p < 0.05 vs. WT, ^#^ *p* < 0.05 vs. WT + DPP4.

To determine whether PAR2 plays a role in human dermal fibrotic disease, PAR2 was knocked down in HDF. To confirm PAR2 expression in knocked-down HDF, the mRNA expression of PAR2 was measured using RT-qPCR (**Figure 7A**). PAR2 mRNA expression was lower in knocked-down HDF than that in wild-type HDF. To investigate whether DPP4-induced fibrotic gene expression in HDF is mediated by PAR2, *ACTA2*, and *COL1A1* mRNA expression in HDF was measured after DPP4 exposure (**Figures 7B, C**). DPP4 induced *ACTA2* and *COL1A1* mRNA expression in HDF, whereas gene knocked-down PAR2 abrogated DPP4-induced fibrotic gene expression (**Figures 7B, C**).

To confirm whether NF-κB and SMAD cause downstream signaling of PAR2, the expression of NF-κB p-p65 (**Figures 7D, E**) and p-SMAD (**Figures 7D, F**) was investigated after DPP4 exposure in HDF. DPP4 induced NF-κB p-p65 and p-SMAD expression in HDF, whereas gene knocked-down PAR2 abrogated DPP4-induced signaling pathways.

## Discussion

Several growth factors and cytokine signaling molecules have been reported to be critical to the activation of cellular mechanisms in fibrotic diseases and the regulation of ECM protein production (Wynn, 2008; Rockey et al., 2015). Soluble DPP4 has physiological and pathological relevance beyond glycemic control. The concentrations of recombinant DPP4 protein used in this study were in the pathological range (Lee et al., 2013). This study observed that soluble DPP4 induced the expression of fibrosis-associated protein in fibroblasts, especially in primary human dermal fibroblasts, suggesting that soluble DPP4 induces the activation of dermal fibroblasts. This result coincides with that of a previous study, which demonstrated that DPP4-positive human dermal fibroblasts express higher levels of myofibroblast markers and collagen in systemic sclerosis (Soare et al., 2020). DPP4 is a functional requirement for fibroblast activation and tissue fibrosis and may serve as an activation marker (Soare et al., 2020).

Studies on the key signaling pathways that regulate fibrosis diseases have reported the following noteworthy findings. TGF-β was observed to be a profibrotic factor and acts as a crucial mediator in fibrogenesis (Walton et al., 2017). The SMAD2/3 intracellular pathway was heavily implicated in TGF-β-induced fibrosis and is known as the canonical pathway (He and Dai, 2015). Targeting the SMAD signaling pathway is a novel therapeutic approach to treating tissue fibrosis (Wojcik et al., 2013; He and Dai, 2015; Liu et al., 2016; Tee et al., 2018; Zhang et al., 2018). In addition to activating the SMAD-dependent pathway, TGF-β can signal in a noncanonical manner, as exemplified in MAPK and NF-κB signaling, which together induce a complete TGF-β response (Wu et al., 2019). The inhibition of a complete TGF-β response may exert beneficial effects that prevent myofibroblast formation and synthesis of ECM components (Luedde and Schwabe, 2011; Madala et al., 2012). NF-κB is the signaling molecule other than SMAD downstream of soluble DPP4. NF-κB acts as a double-edged sword, and the pronounced inhibition may negatively affect cell viability (Gieling et al., 2010). The concentration of the NF-κB inhibitor used in this study did not affect cell viability but abrogated the soluble DPP4–induced fibrotic response. The activation of SMAD and NF-κB signaling may contribute to the fibrotic response of soluble DPP4. Soluble DPP4 and TGF-β exerted different patterns in the activation of the signaling pathway in fibroblasts. In an experimental setting of NIH/3T3 fibroblasts, soluble DPP4 stimulated SMAD and NF-κB signaling, whereas TGF-β stimulated SMAD and ERK signaling. In HDF, both TGF-β and soluble DPP4 induced the activation of p-NF-κB and p-SMAD; in addition, TGF-β induced a more rapid signaling than DPP4 did. The key implication of our findings is that TGF-β possesses stronger and higher potency than soluble DPP4 does on inducing SMAD phosphorylation. To activate SMAD signaling in NIH/3T3 fibroblasts, the requisite concentration of soluble DPP4 is approximately 80–200 ng/ml, whereas the requisite concentration of TGF-β is less than 10 ng/ml.

PAR2 plays crucial roles in tissue hemostasis, thrombosis, wound healing, inflammation-associated disorders, fibrosis, and cancer (Ungefroren et al., 2018). PAR2 activation involves receptor cleavage by different serine proteases and exposure to an N-terminal tethered ligand (TL) that binds to and activates the cleaved receptor (Hollenberg et al., 1997). The activating sequence of PAR2 could be found in the cystein-rich region of DPP4 responsible for binding (Wronkowitz et al., 2014). Therefore, soluble DPP4 can activate PAR2 with its TL sequence and act as an agonist of PAR2 (Wronkowitz et al., 2014). PAR2 has been reported to be involved in soluble DPP4–induced inflammation and dysfunction (Wronkowitz et al., 2014). The blockade of PAR2 prevented soluble DPP4–induced proliferation and inflammation of vascular smooth muscle cells (Wronkowitz et al., 2014) as well as the dysfunction of endothelial cells (Romacho et al., 2016). In addition, PAR2 is expressed on the surface of fibroblasts and has been suggested to play a role in tissue repair processes (Grandaliano et al., 2003; Wygrecka et al., 2011). PAR2 activation induces collagen synthesis and α-SMA expression (Asokananthan et al., 2015). Our results demonstrated that the PAR2 antagonist or knocking down PAR2 can prevent soluble DPP4–induced fibrotic marker expression in fibroblasts, indicating that PAR2 is a receptor of soluble DPP4 and participates in the stimulation of fibroblasts.

The signaling molecules downstream of PAR2 are complex. After being stimulated by soluble DPP4, PAR2 activated the ERK and NF-κB signaling cascades, consequently increasing the secretion of proinflammatory cytokines and stimulating the proliferation of vascular smooth muscle cells (Ervinna et al., 2013; Wronkowitz et al., 2014). Our results indicated that after PAR2 activation, soluble DPP4 stimulated SMAD and the NF-κB pathway to induce a complete response in the activation of fibroblasts because blocking PAR2 using pharmacological inhibitors or genetic knockdown of abolished DPP4 induced SMAD and NF-κB signaling as well as the expression of fibrosis-associated proteins. Membrane-bound DPP4 is essential for TGF-β-induced receptor heterodimerization and subsequent intracellular signal transduction (Shi et al., 2016). The stimulation of cultured dermal fibroblasts with TGF-β induced the upregulation of membrane-bound DPP4 expression (Soare et al., 2020), and the inactivation of DPP4 blocked the TGF-β-induced differentiation of fibroblasts into myofibroblasts and reduced the release of collagen *in vitro* (Soare et al., 2020). Further investigation is required for understanding whether soluble DPP4/PAR2/TGF-β induces a co-activation axis. Studies have reported that inhibition of DPP4 by pharmacological inhibitors alleviated fibrotic responses, such as in bleomycin-induced dermal and pulmonary fibrosis (Soare et al., 2020), CCl_4_-induced liver fibrosis (Kaji et al., 2014; Wang et al., 2017), and a high-salt-diet-induced cardiac failure and fibrosis (Esposito et al., 2017). In our studies, we observed that linagliptin prevented the effect of soluble DPP4 in fibroblasts. Linagliptin can block the interactions between DPP4 and ECM components, receptors, or plasma membrane components (Hasan and Hocher, 2017), thus ameliorating ECM and intracellular signal transduction. The effects of soluble DPP4 on fibroblast activation may be independent of its enzymatic activity.

## Conclusion

**Figure 8:**
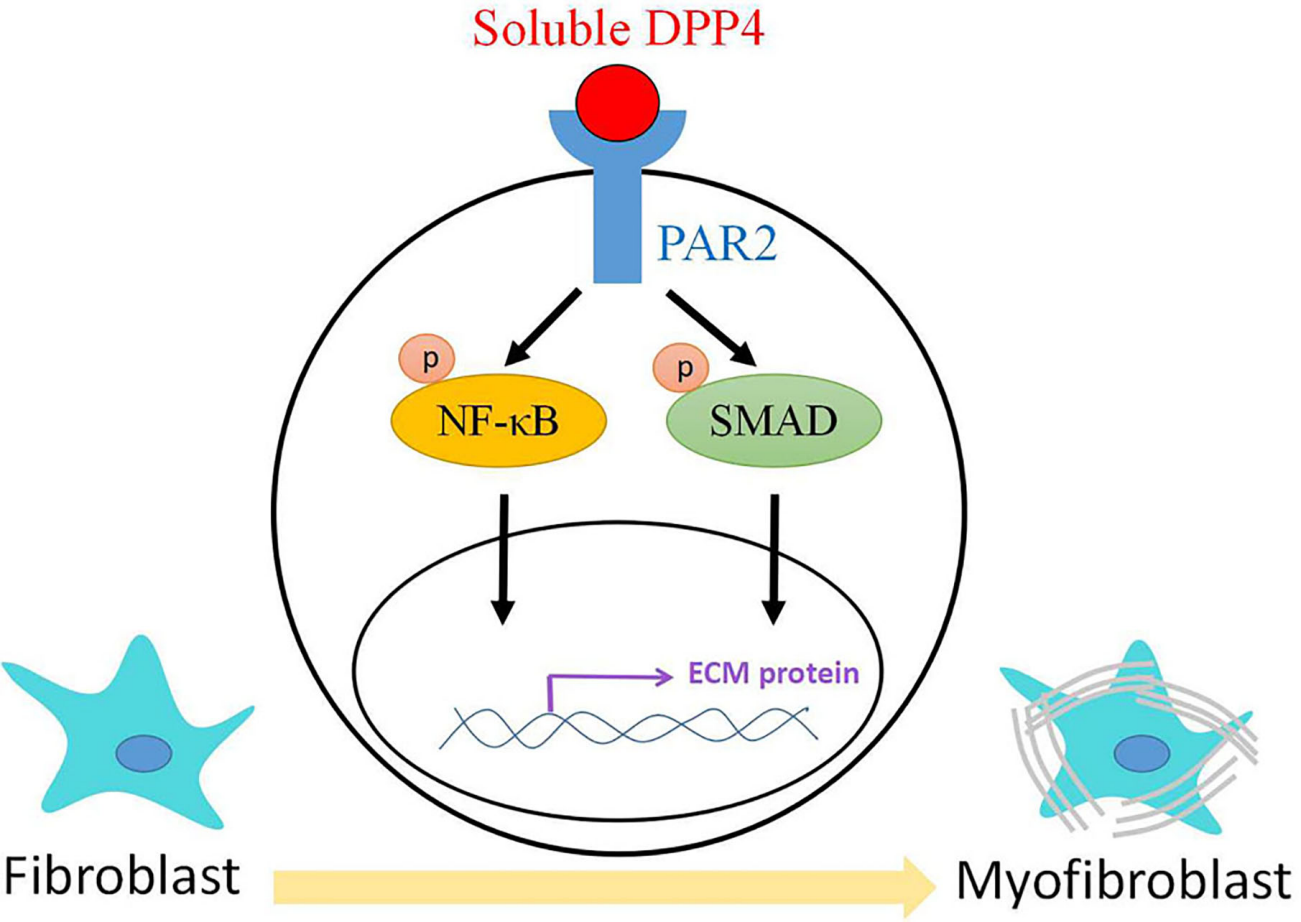
The mechanisms of soluble DPP4.

We characterized the mechanisms underlying soluble DPP4, which activated NF-κB and SMAD signaling through PAR2, leading to the activation of dermal fibroblasts (**Figure 8**). Our data extended the current view of the effect of soluble DPP4 on dermal fibrosis. Elevated levels of circulating soluble DPP4 may contribute to one of the mediators that induce dermal fibrosis in patients.

## Data Availability Statement

The raw data supporting the conclusions of this article will be made available by the authors, without undue reservation.

## Author Contributions

Conceived and designed the experiments: H-CK, S-YL, Y-JL, and M-JS. Performed the experiments: H-CK, S-YL, S-TW, C-WH, and Y-HJ. Analyzed the data: S-YL, S-TW, C-WH, and Y-HJ. Contributed reagents/materials/analysis tools: H-CK, S-YL, and Y-JL. Wrote the manuscript: H-CK and S-YL.

## Funding

We like to thank the research funding from Ministry of Science and Technology, Taiwan (MOST 106-2321-B-030-002-MY3) and (MOST 109-2320-B-030-006-MY3), and Mackay Memorial Hospital, Taiwan (MMH-TT-10907).

## Conflict of Interest

The authors declare that the research was conducted in the absence of any commercial or financial relationships that could be construed as a potential conflict of interest.

## Abbreviations

DCM, Diabetes cardiomyopathy; DPP4, Dipeptidyl peptidase-4; NF-κB, Nuclear factor-kappa B; PAR2, Proteinase-activated receptor-2; ECM, Extracellular matrix; TGF-β, Transforming growth factor-β; α-SMA, α-smooth muscle cell; ERK, Extracellular signal-regulated kinase; NF-κB, Nuclear factor- kappa B; SMAD, suppressor of mothers against decapentaplegic; GAPDH, Glyceraldehyde 3-phosphate dehydrogenase; TL, Tethered ligand.
